# Double-blind, Randomized, Placebo-Controlled study evaluating the Efficacy of an early treatment using Herbal Supplement in the Prevention of Post-Traumatic Stress Disorder in the emergency department (PHYTéS Study)

**DOI:** 10.1192/j.eurpsy.2023.2074

**Published:** 2023-07-19

**Authors:** A. Maktouf Bouhlel, R. Youssef, A. Bouhoula, L. Boukadida, A. Loghmari, K. mansouri, I. Khalifa, H. Yaacoubi, H. Ben Salah, M. Ben othmen, R. Jaballah, A. Zorgati, R. Boukef

**Affiliations:** 1 Emergency Department; 2 Urology, Sahloul teachin Hospital Sousse, Sousse, Tunisia

## Abstract

**Introduction:**

Prevention of Post-traumatic stress disorder (PTSD) is a major public health interestand one of the concerns of any emergency physician.

**Objectives:**

The purpose of this study was to evaluate the efficacy and safety of a herbal supplement in preventing the occurrence of PTSD in high-risk patients.

**Methods:**

: It is a randomized, double-blind, prospective, interventional study including patients exposed to a traumatic event meeting DSM-V Criterion A and having a Peri-traumatic Distress Inventory score and/or Peri-traumatic Dissociative Experience Questionnaire (PDEQ) and/or immediate stress score (L.Crocq score) higher than the thresholds between day 1 and day 3. A total of two hundred patients were included and they were randomly assigned into two groups: Aleozen group and placebo group. Patients included in the aleozen group received Aleozen^®^ for 10 days while patients in the placebo group received a Placebo. A CAPS-5 assessment was performed for all patients at different moments. The main objective was to assess the efficacy of Aleozen^®^ after day 90 of exposure to traumatic events according to PTSD. The secondary objectives were to evaluate the safety of Aleozen^®^ at 10 and 30 days after its administration and to assess PTSD in the involved population after one year.

**Results:**

No statistical differences were noted between the two groups in terms of baseline characteristics, including age, sex, and ISS score. After day 90 of follow-up, and according to the CAPS-5 scale, 85 patients (42.5%) had PTSD. Concerning the primary endpoint, less PTSD was observed in the intervention group compared to the placebo group (38.8% versus 61.2%, respectively; p<0.001). No adverse events were noted during the study

**Conclusions:**

The results of this study suggest the potential preventive effects of an herbal supplement on PTSD for traumatic patients in the emergency department. Further confirmatory studies are required

**Disclosure of Interest:**

None Declared

